# Behavioral Changes and Refractory Hypertension as the First Manifestations of Anaplastic Astrocytoma: A Case Report

**DOI:** 10.7759/cureus.98667

**Published:** 2025-12-07

**Authors:** Julia K Marczuk, Wiktoria M Gembala, Adam T Potocki

**Affiliations:** 1 Department of Primary Care, Jagiellonian University Medical College, Kraków, POL; 2 Family Medicine Clinic, Niepubliczny Zakład Opieki Zdrowotnej "Eskulap-Lubno", Kolczyglowy, POL

**Keywords:** anaplastic astrocytoma, arterial hypertension, behavioral changes, brain glioma, primary care

## Abstract

High-grade brain tumors are uncommon malignancies with poor survival prospects. Their early manifestations can be vague, often resulting in diagnostic delays. Here, we report a case of a 66-year-old woman who exhibited only subtle personality changes and an episode of severe, treatment-resistant hypertension as initial signs of a malignant neoplasm in the brain. These findings prompted neuroimaging, which revealed a large tumor in the right temporal lobe. A biopsy confirmed an anaplastic astrocytoma (WHO grade III). Because of its deep location, surgical removal was not feasible, and radiotherapy was initiated. However, the patient’s condition rapidly worsened due to severe infection, and she died two months after diagnosis. This case underscores the need for vigilance in primary care: even mild behavioral changes or atypical symptoms such as unexplained hypertension in older adults should prompt early investigation for possible brain tumors.

## Introduction

In primary care practice, such nonspecific symptoms can easily be mistaken for internal medicine conditions or psychiatric disorders. Qualitative studies indicate that patients with brain tumors often experience a range of subtle “changes” (e.g., in speech, writing, memory, concentration), which are more often noticed by family members than by the patients themselves [1]. Moreover, such changes frequently lead to loss of interest in activities and withdrawal from daily life [1]. Classic symptoms, such as seizures, may not appear in some patients [1], which further complicates early recognition.

Anaplastic astrocytoma (astrocytoma anaplasticum) is a malignant brain tumor classified as a WHO grade III glioma. It accounts for approximately 1.7% of all central nervous system neoplasms and 6-7% of gliomas in adults [2].

Typically, it presents similarly to other brain tumors - with headaches, seizures, neurological deficits, or signs of increased intracranial pressure. However, the initial manifestations may sometimes be nonspecific and involve the psychological domain, such as mood changes, cognitive disturbances, or personality alterations [3]. In patients presenting with behavioral changes, the differential diagnosis must extend beyond primary psychiatric disorders. Organic causes, such as metabolic disturbances (e.g., hyponatremia, hepatic encephalopathy), endocrinopathies (e.g., Cushing’s syndrome, hyperthyroidism), intracranial neoplasms, infections (e.g., neurosyphilis, HIV), and autoimmune conditions (e.g., N-methyl-D-aspartate (NMDA) encephalitis, systemic lupus erythematosus (SLE)), should be considered early in the diagnostic process. A structured approach involving corroborative history, targeted physical examination, and basic laboratory and imaging workup is essential to avoid misattributing such symptoms to functional disorders. As emphasized by Wang et al., organic etiologies often mimic psychiatric presentations and may lead to delayed recognition of serious underlying disease [4].

Neurological symptoms associated with brain tumors are often subtle and nonspecific, making early detection particularly challenging. Behavioral shifts, decreased engagement in daily routines, and gradual social withdrawal may be overlooked or misattributed to functional disorders. Furthermore, the absence of classic manifestations such as seizures can delay appropriate workup [1]. In the case presented, it was the unusual behavioral changes and treatment-resistant hypertension that served as early warning signs, ultimately leading to the diagnosis of an anaplastic astrocytoma.

## Case presentation

The patient, a 66-year-old woman, non-smoker with no significant chronic diseases in her medical history. Until recently, she had been very physically and socially active; her family described her as meticulous and well-groomed.

Onset of symptoms: At the beginning of 2021, the patient’s relatives noticed unusual changes in her behavior. Over the course of several weeks, she became apathetic, withdrawn, and less engaged in daily activities. She stopped maintaining order at home and neglected her appearance, which contrasted sharply with her previously meticulous personality. During meetings of the Nordic walking group she attended, she changed from being a leading participant to someone who lagged behind. The patient’s daughter noticed that her mother began dressing carelessly, for example, buttoning her clothes unevenly or wearing a crooked hat, which she had never done before. No classic symptoms of depression or psychosis were observed - the change affected mainly her personality and daily habits. The initial course was atypically paucisymptomatic - neither paresis, nor aphasia, nor seizures were noted. Only later did subtle neurological symptoms appear, such as slight tripping of the left leg, weakness of the left arm, and possible visuospatial perceptual disturbances. In mid-February 2021, the patient presented to her primary care physician due to increasing headaches and general deterioration.

Hypertensive crisis episode: On February 27, 2021, during the night hours, the patient presented to out-of-hours care with severe, diffuse headache and a spike in blood pressure to 199/122 mmHg (pulse 96/min). Despite administration of as-needed nifedipine, furosemide, hydroxyzine, and metoprolol, her blood pressure only partially decreased (to 154/97 mmHg). Physical examination did not reveal any neurological deficits or disturbances of consciousness. A hypertensive crisis was diagnosed, assumed secondary to primary hypertension, and continuation of antihypertensive therapy was recommended. Follow-up laboratory tests (March 1, 2021) showed no significant abnormalities: complete blood count, electrolytes, glucose levels, and urinalysis were within normal ranges (only total cholesterol at 203 mg/dL slightly exceeded the reference values).

Further follow-up by the primary care physician: The primary care physician paid particular attention to the unusual change in the patient’s functioning - during visits, she appeared unusually calm, with a “glassy” look in her eyes. Lack of improvement with standard antihypertensive treatment and unclear neurological symptoms prompted the physician to broaden the diagnostic workup.

**Figure 1:**
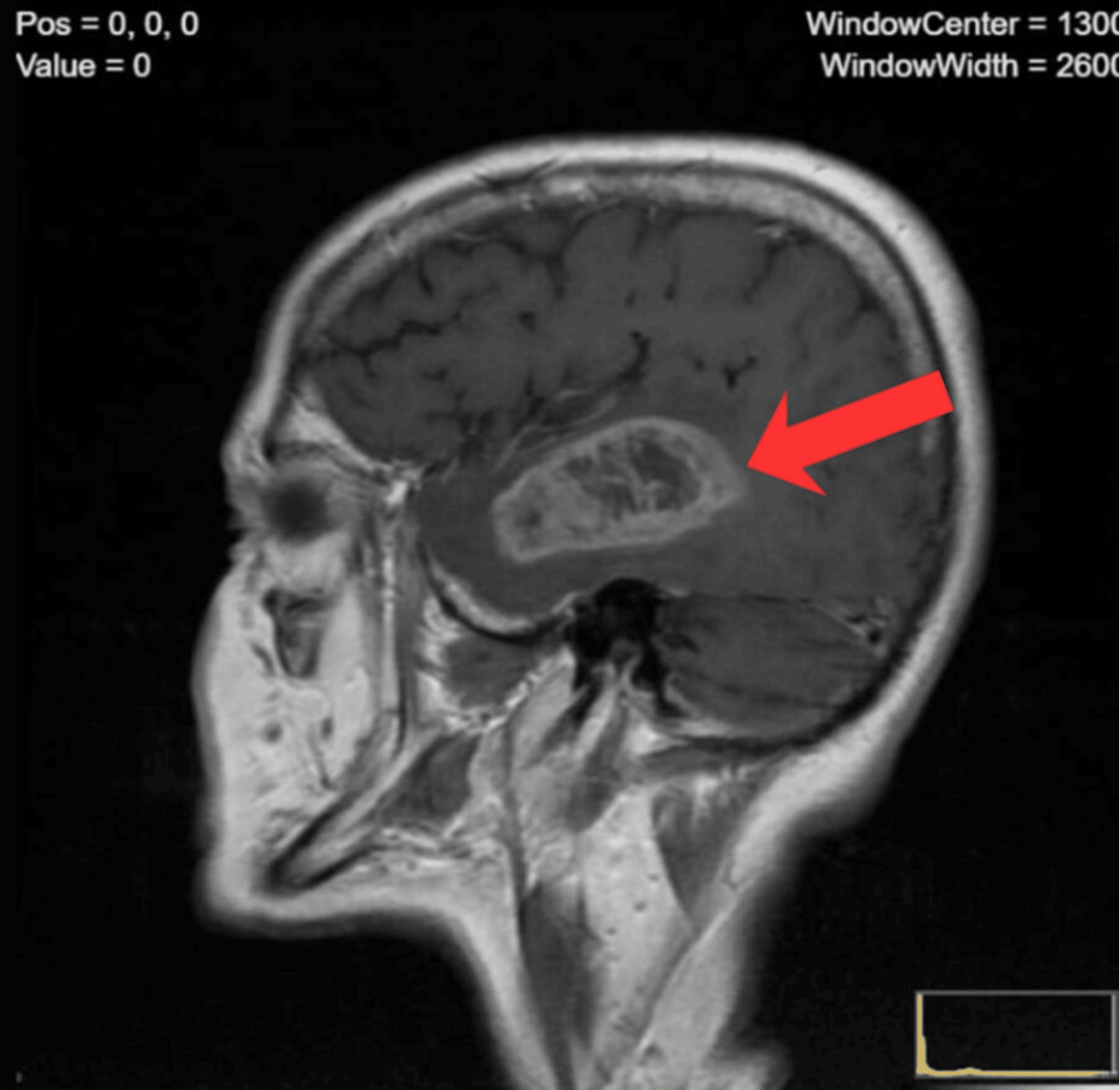
Sagittal contrast-enhanced MRI of a right temporal lobe mass Sagittal contrast-enhanced MRI demonstrating a large heterogeneous mass in the right temporal lobe with irregular ring enhancement and central necrosis. The lesion invades deep cerebral structures and produces a marked mass effect on the adjacent parenchyma. Surrounding vasogenic edema and compression of the right lateral ventricle are evident.

**Figure 2:**
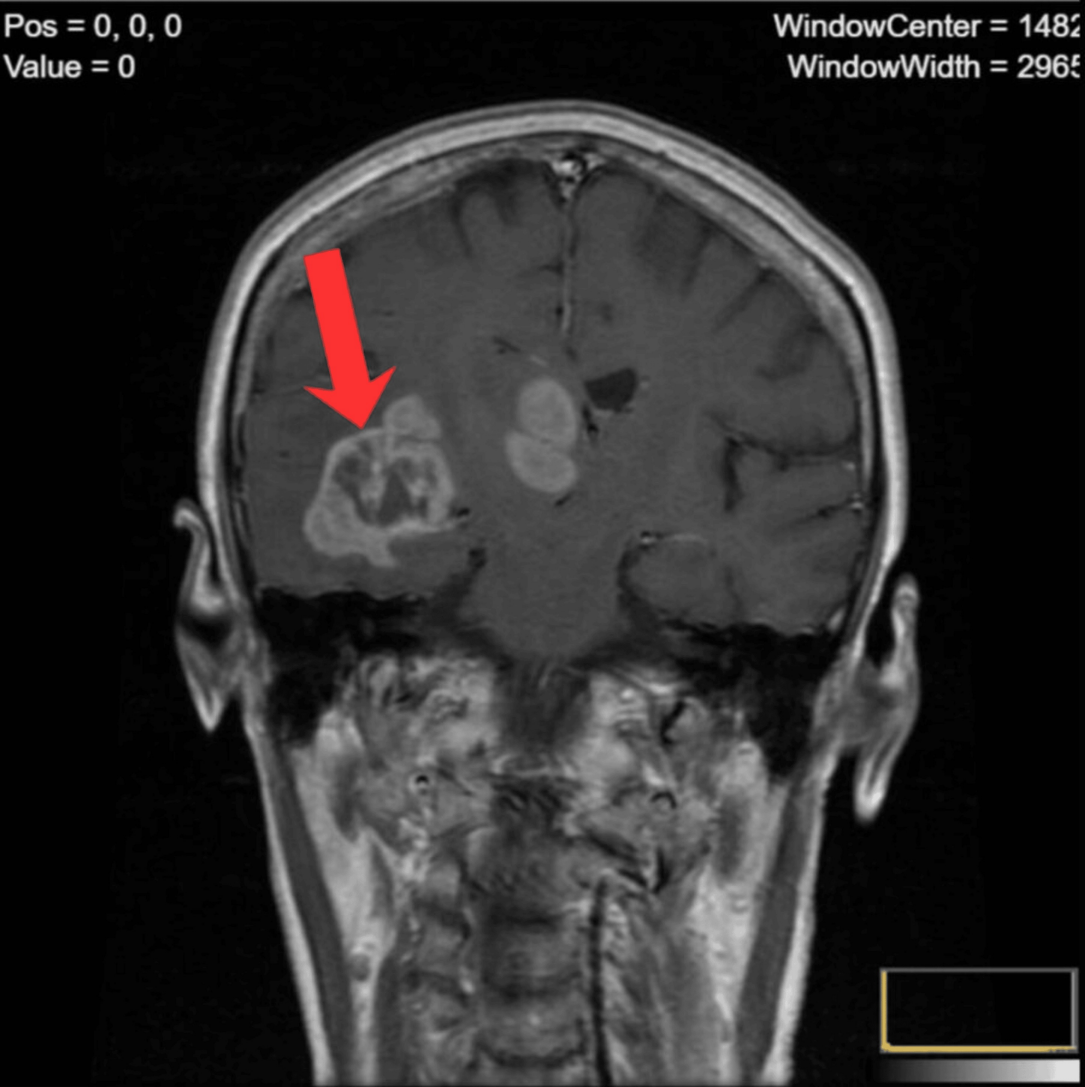
Coronal contrast-enhanced MRI of a right temporal lobe mass Coronal contrast-enhanced MRI demonstrating a large irregularly enhancing mass within the right temporal lobe, accompanied by a second adjacent enhancing lesion. The dominant tumor exhibits ring-like enhancement with central necrosis and surrounding vasogenic edema, producing significant mass effect on midline structures.

Imaging diagnostics: A neurology consultation (routine) and urgent central nervous system (CNS) imaging were ordered. The family decided to obtain a private brain MRI to shorten the waiting time. Contrast-enhanced MRI performed on March 10, 2021, revealed an extensive tumor mass in the right cerebral hemisphere - primarily located in the temporal lobe and extending deeper into central structures (Figure 1). The tumor measured approximately 67 × 54 × 34 mm and exerted a marked mass effect on surrounding tissue (Figure 2). Upon receiving the imaging results, the patient was urgently referred to a neurosurgical center and then to an oncology center for further management.

Histopathological diagnosis: On March 24, 2021, a stereotactic biopsy of the brain tumor was performed at the neurosurgical center. Tissue samples obtained for histopathological examination confirmed the diagnosis of an anaplastic astrocytoma (astrocytoma anaplasticum) - a malignant WHO grade III glioma. Molecular studies showed methylguanine-DNA methyltransferase (MGMT) promoter methylation, absence of 1p/19q codeletion, and no IDH1/IDH2 mutations (IDH-wild type). Post-procedure, the patient was treated with dexamethasone to reduce cerebral edema, which temporarily improved symptoms of intracranial pressure.

Treatment and clinical course: Due to the location of the tumor (deep structures of the dominant hemisphere), radical neurosurgical resection was deemed too high-risk. A multidisciplinary oncology board planned combined treatment with radical radiotherapy. On May 15, 2021, the patient was admitted to the Radiotherapy Department for treatment planning. Her general condition upon admission was relatively stable; steroid therapy (dexamethasone) was continued to prevent progression of intracranial hypertension symptoms. Laboratory tests at admission revealed only mild thrombocytopenia (133 × 10³/µL) and hyponatremia (133 mmol/L. Radiotherapy using IMRT was initiated (planned: 15 fractions of 2.67 Gy, total dose of 40.05 Gy to the tumor area). The patient initially tolerated the treatment well.

**Figure 3:**
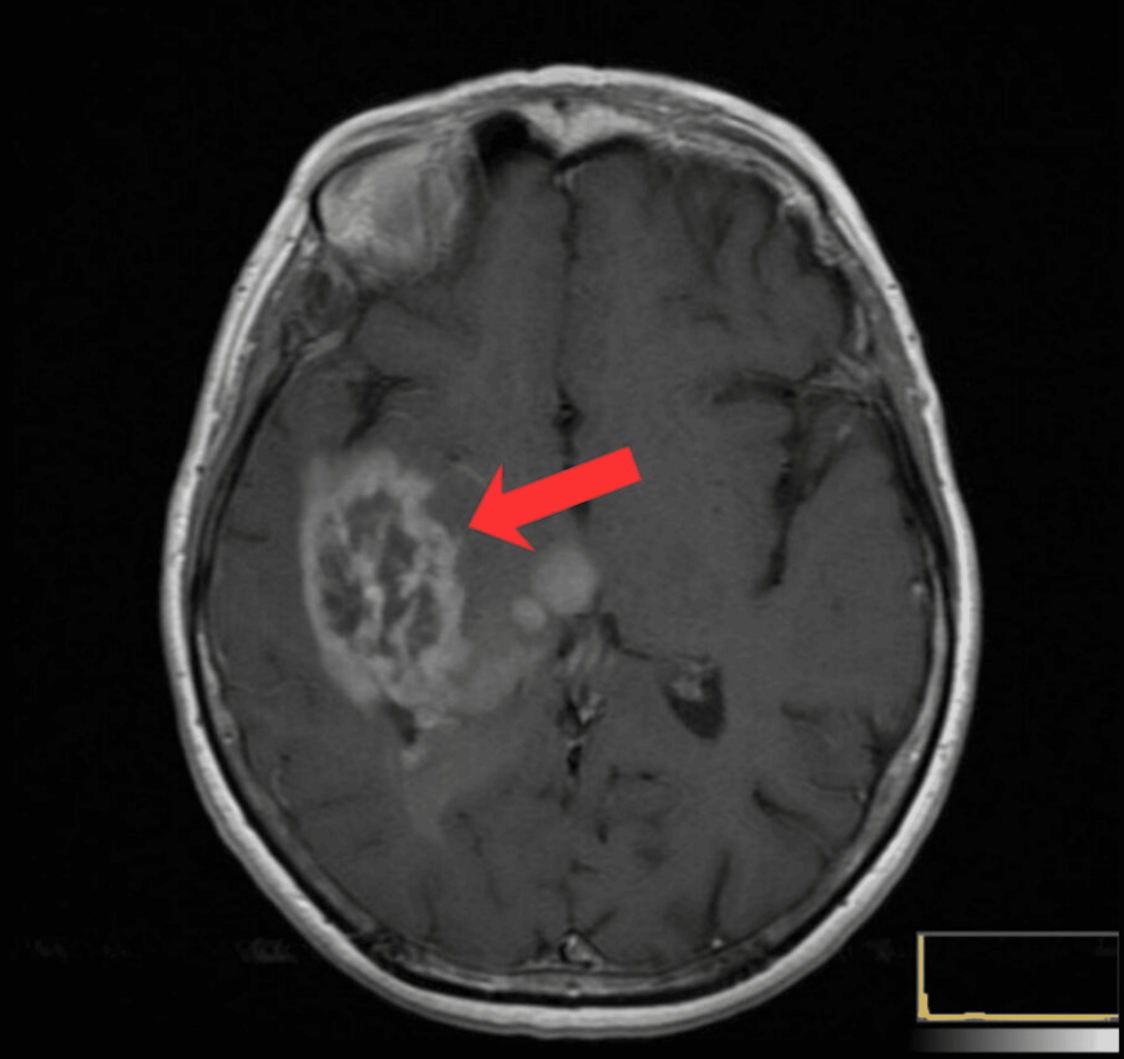
Axial contrast-enhanced MRI of a right temporal lobe mass Axial MRI demonstrating a large, heterogeneously enhancing mass in the right temporal lobe, characterized by a thick, irregular ring-enhancing rim and central necrosis.

Complications: Around May 25, 2021, signs of systemic infection appeared-fever and chills. Procalcitonin levels increased to 0.88 ng/mL, indicating the onset of severe infection. Empirical antibiotic therapy was initiated. On May 26, a right basilic-vein PICC line was inserted to provide stable venous access. Despite intensive treatment, the following day the patient’s condition deteriorated rapidly - sepsis developed (procalcitonin 20.9 ng/mL), along with a sudden rise in blood glucose (19.49 mmol/L, ~351 mg/dL). The patient was treated in the ICU with aggressive fluid resuscitation, intravenous antibiotics, and insulin. Unfortunately, during hospitalization, she experienced sudden cardiac arrest and died on May 28, 2021, approximately two months after diagnosis. Figure 3 presents the axial contrast-enhanced MRI of a right temporal lobe mass.

## Discussion

The presented case illustrates the diagnostic challenges posed by brain tumors with atypical presentation in primary care practice. The literature emphasizes that early symptoms of CNS neoplasms are often non-specific and easily mistaken for other conditions [1]. Walter et al. noted that patients with brain tumors frequently presented to their primary care physician several times before diagnosis due to various “minor” complaints or changes in functioning rather than a single clear symptom [1]. In our patient, such a subtle prodrome was a behavioral change: apathy, neglect of daily activities, and reduced physical activity. The family described her as “a different person” than before. The literature confirms that personality or behavioral changes may be the first sign of a brain tumor - according to various sources, they occur in a substantial proportion of patients with gliomas (from approximately 8% up to even 67% of cases, depending on the studied population and definition criteria) [5]. Importantly, these changes are often gradual and initially subtle, which means they may remain unnoticed until a more advanced stage of the disease [6]. The brain, due to neuroplasticity, can compensate for the presence of a growing tumor for some time, resulting initially in only subtle symptoms. In our case, none of the typical neurological manifestations (e.g., weakness, aphasia, seizures) occurred at the beginning - only later did mild neurological symptoms appear (tripping over the left leg, weakness of the left arm, and possible visuospatial disturbances), when the tumor had already reached a large size. According to reports, not every patient with a brain tumor presents with seizures - approximately 20-30% of patients with brain tumors do not experience seizures at all [1]. In our patient, no seizures were observed, which aligns with these observations.

Attention should be drawn to the unique element of this case - the episode of hypertensive crisis that preceded the diagnosis. A sudden spike in blood pressure resistant to standard treatment is an unusual symptom in the course of a brain tumor. Several mechanisms may potentially explain this phenomenon. Increased intracranial pressure caused by the presence of a large tumor may trigger the so-called Cushing reflex - a physiological compensatory reaction in which there is a rapid increase in arterial blood pressure in response to ischemia of the vasomotor center in the medulla oblongata. Harvey Cushing, already at the beginning of the 20th century, described the triad of symptoms of increased ICP: hypertension, bradycardia, and respiratory irregularities [7]. In our patient, the heart rate was 96/min (bradycardia did not occur); nonetheless, the dramatic elevation in systemic blood pressure could have resulted from such a reflex in the context of severe headache and rising intracranial pressure. Another possible explanation is the potential location of the tumor or edema near hypothalamic-brainstem structures involved in autonomic regulation. Rare cases have been described in the literature in which posterior fossa tumors (e.g., cerebellar hemangioblastoma) caused malignant hypertension due to compression of the brainstem, which resolved after tumor removal. Cameron and Doig, as early as 1970, described two such situations where a cerebellar tumor mimicked the symptoms of a pheochromocytoma (paroxysmal hypertensive crises) [8]. In our case, the tumor was located supratentorially (temporal lobe), making the mechanism of direct brainstem compression unlikely. Nevertheless, extensive cerebral edema and increased ICP may have triggered a transient hypertensive response. This episode drew the attention of the primary care physician and prompted further diagnostic pursuit, although it was initially classified as a hypertensive crisis of unclear etiology.

MRI imaging revealed a very large tumor (approx. 6-7 cm in diameter) in the right temporal lobe (Figure 3). Such a substantial size with relatively sparse neurological symptoms may result from slow tumor growth and brain compensation. Lower-grade gliomas (II-III) grow more slowly than glioblastoma, allowing the brain time to adapt. Unfortunately, biopsy ultimately confirmed a malignant neoplasm - an anaplastic astrocytoma, a WHO grade III glioma. In the 2016 WHO classification, anaplastic astrocytomas were differentiated into IDH-mutant and IDH-wildtype; the latter, lacking the IDH mutation, had a significantly worse prognosis (behaving like glioblastoma) [9]. In our patient, no IDH mutation was found, which explains the aggressive course of the disease - in fact, IDH-wildtype gliomas with anaplastic features are now classified as glioblastoma (WHO IV) due to similar biology and prognosis [10]. On the other hand, MGMT promoter methylation was present, which is a favorable predictive factor for response to alkylating chemotherapy (temozolomide) [9]. Unfortunately, our patient did not survive to begin chemotherapy - she died before treatment could be initiated due to infectious complications. Optimal treatment for anaplastic astrocytoma involves maximal safe surgical resection followed by radiotherapy and temozolomide chemotherapy [9]. According to statistics, the average survival of patients with anaplastic astrocytoma (overall) is approximately five years [9]; however, this applies mainly to patients with IDH-mutant tumors, younger individuals, and those in good general condition. Patients with IDH-wildtype, inoperable tumors, or of older age have much worse prognoses - similar to grade IV gliomas (median survival 12-15 months or less). Our patient belonged to a high-risk group: age 66, inability to undergo tumor resection, and tumor biology indicating high malignancy. Her rapid deterioration and death only two months after diagnosis confirm the aggressive nature of the disease.

From the primary care physician’s perspective, the presented case conveys an important lesson. The patient’s multiple visits for seemingly minor problems (worsened well-being, difficult-to-control hypertension, unusual behavior) were opportunities to detect “red flags.” A holistic approach proved crucial - speaking with the family and observing changes in the patient’s functioning. Reports in the literature suggest that repeated subtle changes in a patient’s condition, noticed by relatives, often precede the diagnosis of a brain tumor [1]. A primary care physician, who knows the patient well, is uniquely positioned to detect such deviations from baseline. In the discussed case, rapid referral for imaging (despite the absence of focal neurological deficits) enabled relatively early detection of a large tumor. Unfortunately, disease advancement and lack of radical treatment options determined the poor outcome. One may speculate that had the patient undergone MRI even earlier - e.g., at the onset of the first behavioral changes - the tumor might have been smaller, potentially allowing for surgical intervention and improved prognosis.

## Conclusions

The described case highlights the importance of oncologic vigilance in primary care. Even subtle behavioral changes in an older adult, especially when combined with an atypical course of chronic conditions (e.g., hypertension resistant to treatment), should prompt the physician to broaden the diagnostic workup. Early identification of a brain tumor offers the possibility of earlier initiation of oncologic treatment and potentially better therapeutic outcomes. Furthermore, this case underscores the complexity of the clinical presentation of gliomas-psychiatric or nonspecific symptoms may precede the emergence of classic neurological signs. Interdisciplinary collaboration between the primary care physician, neurologist, and oncologist is crucial for the prognosis of a patient with an ambiguous clinical picture.
